# Detection of the Crystallization Process of Paracetamol with a Multi-Mode Optical Fiber in a Reflective Configuration

**DOI:** 10.3390/s20010087

**Published:** 2019-12-22

**Authors:** Liliana Soares, Susana Novais, António Ferreira, Orlando Frazão, Susana Silva

**Affiliations:** 1 INESC TEC—Institute for Systems and Computer Engineering, Technology and Science, Rua do Campo Alegre 687, 4169-007 Porto, Portugalsusana.novais@inesctec.pt (S.N.); ofrazao@inesctec.pt (O.F.); 2 Faculty of Biotechnology of Catholic University of Portugal, Rua de Diogo Botelho, 1327, 4169-005 Porto, Portugal; 3 LEPABE—Laboratory for Process Engineering, Environment, Biotechnology and Energy, Department of Chemical Engineering, Faculty of Engineering, University of Porto, Rua Dr. Roberto Frias s/n, 4200-465 Porto, Portugal; amaf@fe.up.pt; 4 Department of Physics and Astronomy, Faculty of Sciences of University of Porto, Rua do Campo Alegre 687, 4169-007 Porto, Portugal

**Keywords:** paracetamol, crystallization, multi-mode optical fiber

## Abstract

A configuration of a refractometer sensor is described with the aim of optically detecting the crystallization process of paracetamol. The developed sensing head is based on a conventional cleaved multi-mode fiber. The fiber tip sensor structure was submitted to contact with the liquid of interest (paracetamol fully dissolved in 40% *v*/*v* of ethanol/water) and the crystallization process of paracetamol, induced with continued exposure to air, was monitored in real time.

## 1. Introduction

Crystallization is one of the processes considered oldest when used for purification and separation of solid products. In most cases, it allows a solid product to be obtained from a liquid solution, however, it can occur from a molten mixture or even a gas, always involving a change of phase. This process is commonly used by most industries including food, microelectronics, bulk, and fine chemicals and pharmaceuticals.

Regarding processes of crystallization of active pharmaceutical ingredients (APIs), usually the most critical and least understood are those that have multiple polymorphic forms. Often, process and product errors result due to a lack of understanding and poor control of crystallization methods [1,2]. The API could be observed as the primary stage in the formulation process, with molecules stabilized within the crystal lattice during the following processing phases, until the crystal dissolves upon administration to the patient permitting the molecular form of the drug to be absorbed [3].

The first stage—when the pure solid product is separated from the liquid—is crystallization, and this process is considered crucial to adapt the solid properties like shape, polymorphic form, crystal size distribution, and purity. Since the previously mentioned characteristics play a crucial role in the properties and the quality of the final product, such as dissolution behavior, shelf life or bioavailability, as well as in processes such as drying or filtration, it is important to have an appropriate design and control of crystallization methods [1,3].

Throughout the crystallization process, there are two consecutive steps, namely, nucleation and crystalline growth, and based on these steps, significant efforts have been concentrated during the last past decades, to better understanding these mechanisms [4,5,6]. That said, a strong investment in research has been made on the control and modeling of crystallization systems, with great progress essentially due to the development and broader applications of process analytical technology (PAT) tools, allowing the increase in computing power [1].

The continuous development of new cost-effective PAT tools is crucial. Several low-cost sensors have been proposed for in situ process monitoring, such as dielectric constant measurements [7,8], refractive index, acoustic methods [9,10], and acquisition of optical images [11]. Boerkamp et al. propose for scale formation monitoring an exposed core optical fiber sensor [12]. Besides, in future, the non-contact sensors will obtain substantial attention in, for instance, external imaging [13] and external acoustic monitoring [14].

Paracetamol is commonly classified and used as an analgesic and antipyretic and is a compound well-characterized and reported in the literature. It is composed of three polymorphs: stable form I (monoclinic), metastable form II (orthorhombic), an unstable form III [15]. Their stability at standard temperature and pressure can be defined as form I > form II > form III [16]. Supercooling the melt and preparing an amorphous form, which is the least stable of all its solid forms, can be considered a simple process [17,18]. Taking account of the three polymorphs identified, the monoclinic crystal (form I) is considered the more stable at room conditions [19]. Nevertheless, below −120 °C, form II polymorph becomes more stable than form I [20].

Due to minor experimental conditions, the paracetamol, despite its well characterized solid forms, displays peculiar crystallization behavior [15,21]. In 2012, Nanubolu et al. developed a work which demonstrated a large comparison between several experimental conditions and crystallization patterns [22]. It was concluded that the enclosed samples were dominated by bulk crystallization into form III and the disclosed samples were outweighed by surface crystallization into forms I and II.

Regarding paracetamol crystallization, it was important to conduct a study survey on the characterization of polymorphs and crystallization process monitoring. In different studies, it employed the Fourier transform Raman spectroscopy for a fast and simple quantitative analysis method of the polymorph mixtures in paracetamol powder [20,21,22,23], whereas the Raman and Fourier transform infrared spectroscopy (FTIR) were reported to be proper for the quantitative determination of forms I and II in a powder mixture based on a linear correlation [24]. Other studies revealed that Raman detected differential scanning calorimetry (DSC) was used to monitor the DSC thermos diagrams during the supercooling of paracetamol. When Raman and DSC were joined for characterization, thermally driven polymorphic transitions were allotted unambiguously [25].

In this work, a simple configuration of a refractometer sensor based on a conventional cleaved multi-mode fiber tip was implemented to monitor in real time the paracetamol crystallization process by means of intensity variation based on the fiber tip–interaction concept.

## 2. Multi-mode Fiber-Based Tip Sensor Characterization

**Figure 1:**
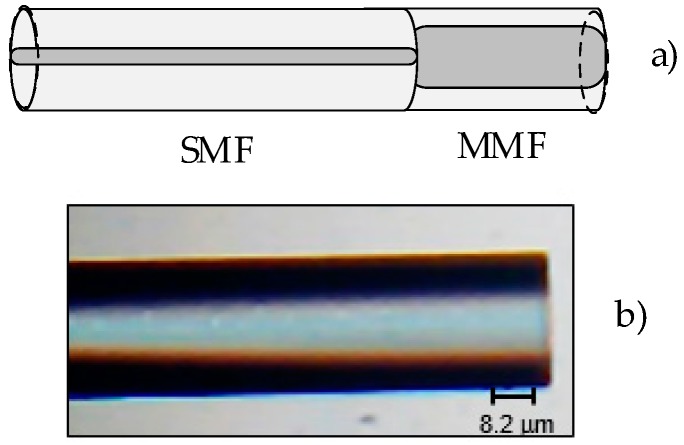
Schematic diagram of the sensor structure (**a**) and a microscope image (**b**).

The proposed sensor tip is based on a conventional multi-mode fiber (MMF GIF 625, supplied by Thorlabs, Newton, NJ, USA), with core and cladding diameters of 62.5 µm and 125 µm respectively, spliced to a single-mode fiber (SMF 28e, supplied by Thorlabs, Newton, NJ, USA) (see Figure 1). The end of the multi-mode fiber (MMF) was then cleaved, in order to obtain Fresnel reflection at the fiber tip. The MMF tip configuration was chosen as to ensure a higher amount of the reflected optical signal due to the larger area of the core. The operating mechanism of the fiber probe relies on the measurand-induced intensity variation of the Fresnel reflection at the fiber-to-liquid interface monitored at a selected spectral window. Upon reaching the surroundings, the light is partially reflected. In this way, the obtained optical spectrum is the result of the reflected wave and the measurement of refractive index variations is achieved by intensity changes of the reflected optical signal.

Considering that optical monitoring of paracetamol concentration using a reflective fiber optic refractometric sensor is the main objective of the present work, the sensitivity of the developed optical sensor to the refractive index was tested using liquid samples of paracetamol with different concentrations. A series of mixtures consisting of different mass fractions of paracetamol in ethanol/ deionized water (40% *v*/*v*) were previously prepared under a controlled laboratory environment at room temperature of 23 °C. A magnetic stirrer was used to prepare the samples with a concentration range of ~50 to 260 g/kg, corresponding to a refractive index range of 1.3634 RIU and 1.3947 RIU. For the calibration of said solutions, the refractive index was determined for all the samples using an Abbe refractometer.

**Figure 2:**
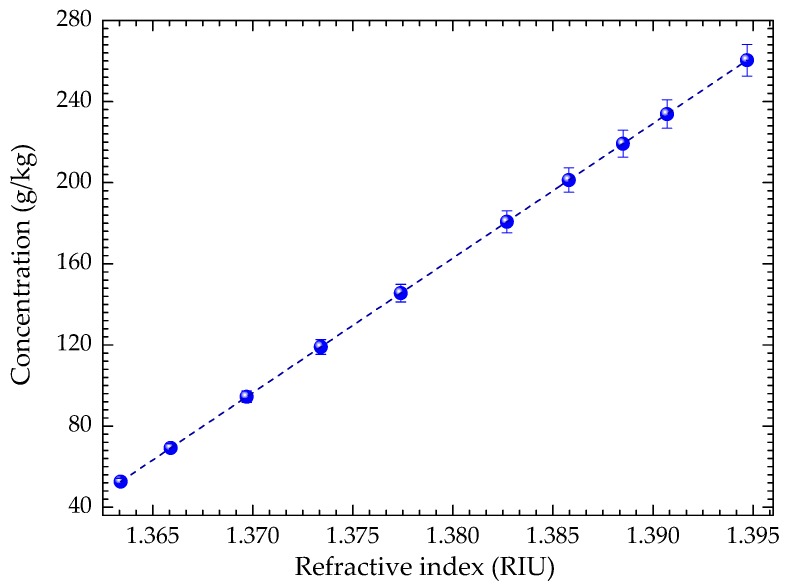
Paracetamol concentration dependence on the refractive index.

As shown in Figure 2, a linear relationship was obtained between the paracetamol concentration and the measured refractive index. This result indicates that with the increase in paracetamol concentration, the samples become optically denser and consequently, its refractive index increases.

**Figure 3:**
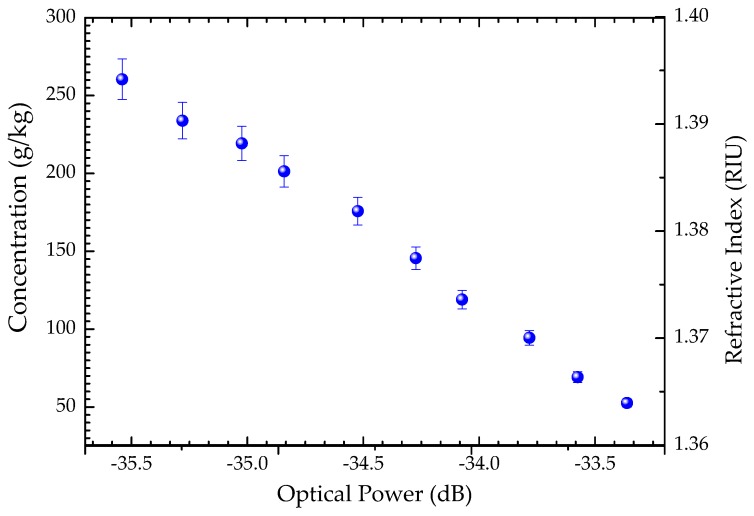
Optical power dependence on paracetamol concentration and respective refractive index.

After liquid sample calibration, the sensitivity of the MMF-based tip sensor to the refractive index changes was determined, using the previously prepared paracetamol liquid samples with distinct concentrations. The experimental setup used in the experiment corresponds an 80 nm-wide broadband optical source, centered at 1550 nm, connected to the optical spectrum analyzer by means of an optical circulator. The sensing head was immersed vertically in each of the prepared paracetamol samples. Figure 3 illustrates the behavior of the proposed sensor to the measured parameters. The experimental data was well adjusted for a linear function (with a correlation factor of 0.989). The reflected optical power decreases with increasing paracetamol concentrations, as the liquid surrounding medium becomes optically denser, the refractive index increases.

From the results presented in Figure 3, a linear sensitivity to paracetamol concentration of −10.15 ± 0.35 dB/(g/g) was achieved, within ranges from ~50 to 260 g/kg and corresponding refractive index sensitivity of −67.36 ± 2.35 dB/RIU, within the ranges of 1.3634 RIU and 1.3947 RIU.

**Figure 4:**
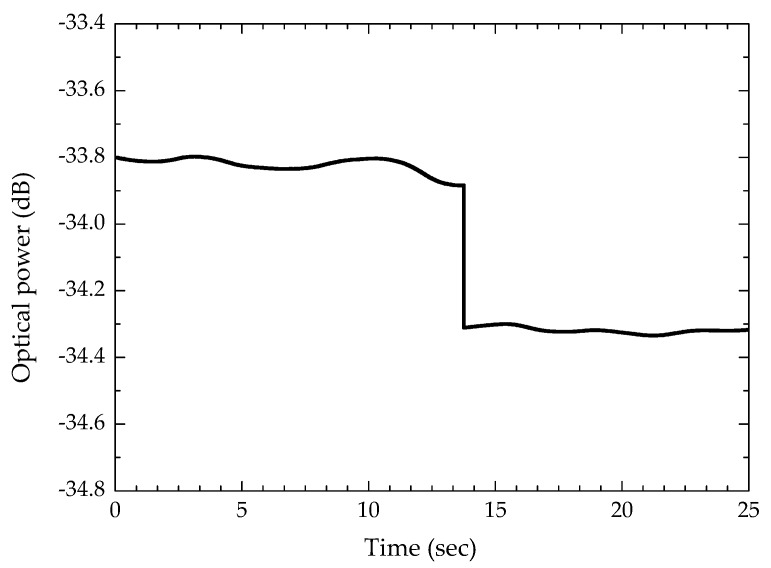
Step technique to estimate the resolution of the sensor.

Concerning the stability of the projected sensor, preliminary tests were also carried out using the experimental setup referred for the sensitivity evaluation and two samples of paracetamol with consecutive refractive indices. The sensing head was consecutively immersed in the two paracetamol samples, obtaining the response shown in Figure 4, for a measurement wavelength of 1550 nm. The minimum value of the refractive index *δ_n_* that the sensor can discriminate is given by Equation (1) [26]:

(1) $$\delta_{n}=2\frac{\sigma_{p}\Delta_{n}}{\Delta P}$$

where *σ_p_* is the maximum standard deviation of the optical power for both values of refractive index (1.3659 and 1.3734), and Δ*_n_*, Δ*P* are the variation of refractive index (7.5 × 10^−3^ RIU) and the mean displacement of optical power between the two steps, respectively. It is noted that the refractive index variation (7.5 × 10^−3^ RIU) corresponds to a paracetamol concentration variation of 49.79 g/kg. By applying Equation (1), a resolution of 5.3 × 10^-4^ RIU was obtained, corresponding to a resolution of 3.53 × 10^−3^ g/g, respectively (see Figure 4). It is important to note that this value is also influenced by the spectral resolution of the equipment used for data acquisition.

## 3. Monitoring the Crystallization Process of Paracetamol

The monitoring of the paracetamol crystallization process was performed by analyzing the response of the MMF-based tip sensor in terms of intensity variation of the optical signal interrogated in reflection.

**Figure 5:**
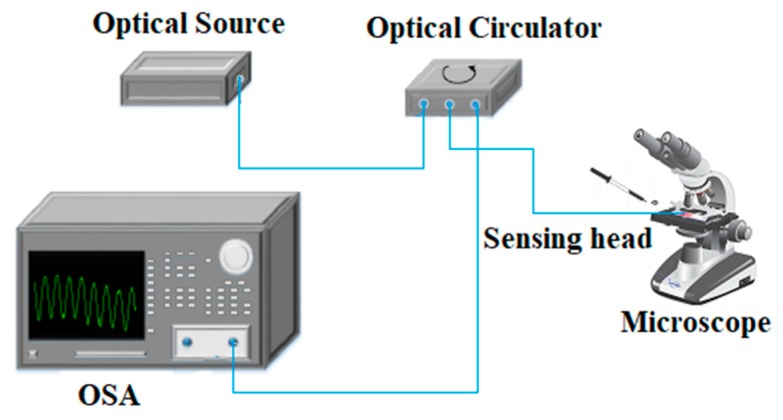
Experimental setup for paracetamol crystallization process monitoring.

The schematic of the experimental setup is presented in Figure 5. An 80 nm-wide broadband optical source centered at 1550 nm was connected to the optical spectrum analyzer (OSA) using an optical circulator, and the sensing head was fixed to a coverslip to monitor the crystal formation process microscopically.

The refractometric fiber tip was placed horizontally on a coverslip and with the aid of a pipette, a drop was spilled onto the sensor, as shown in Figure 5. Notice that the sensing head was also fixed to the coverslip, providing sensor robustness as well as the stability of the fiber. Further, a drop of the previously prepared paracetamol sample (with a concentration of 200 g paracetamol/kg solvent, in an ethanol/water mixture, 40% *v*/*v*) was deposited on the sensing structure as to ensure that it was fully in contact with the liquid solution.

**Figure 6:**
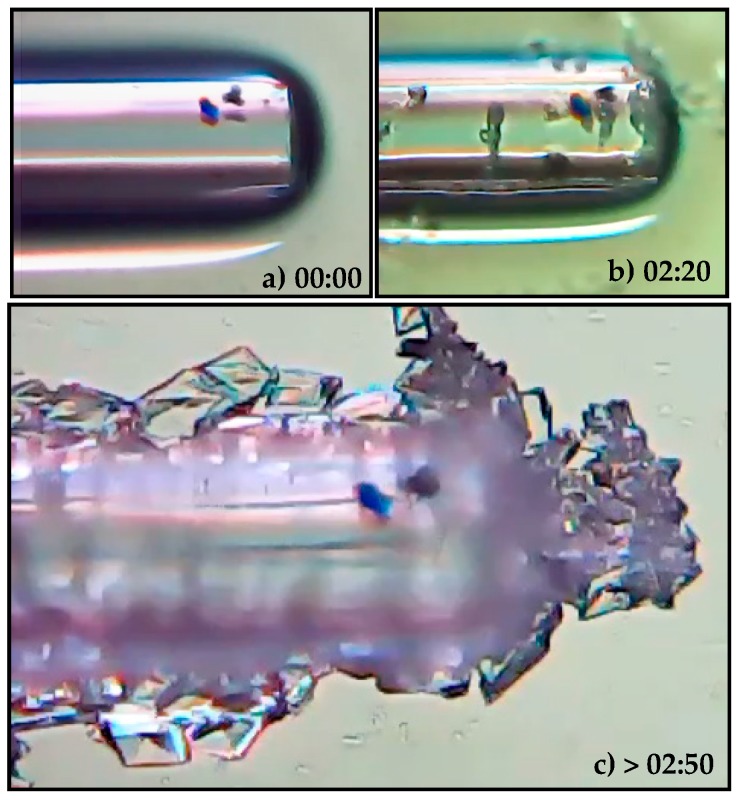
Crystallization process of paracetamol (video in Supplementary Materials) (**a**) Liquid paracetamol solution in contact with the MMF-tip sensor, (**b**) beginning of the paracetamol crystallization with continuous exposure to air and (**c**) full evaporation of the liquid solution and formation of crystalline structures of paracetamol.

The crystallization process of paracetamol was induced by continued exposure to air, which led to the evaporation of ethanol and the consequent increase in supersaturation. In Figure 6 it is possible to observe the crystalline structures formed around the sensing head, verifying that they are essentially monoclinic polymorphs (video in Supplementary Materials). At room temperature, this form of paracetamol crystal (form I) is considered the more stable at room temperature conditions [19].

**Figure 7:**
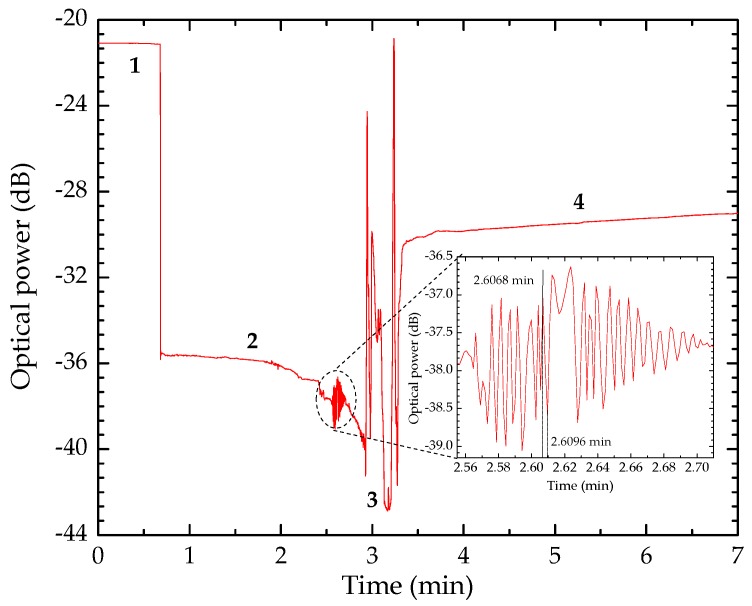
Monitoring of the crystallization process. (1) Addition of the paracetamol sample; (2) air exposure; (3) initiation of crystallization process; (4) stabilization, (zoom in between the steps (2) and (3)).

Signal variation throughout the crystallization process, at a measuring wavelength of 1550 nm and an acquisition rate of 700 Hz, is shown in Figure 7, where (1) corresponds to the addition of paracetamol to the coverslip containing the sensing head, (2) to the onset of air exposure, (3) to the beginning of the formation of crystalline structures and (4) the stabilization and termination of the crystallization process, in which the sensing head is surrounded by paracetamol crystals (see Figure 6). It is important to note that in phase 4 the crystals are still wet, which leads to slight instability by the sensor. It is also worth notice that the presence of the fiber tip sensor accelerates the crystallization process due to the capillarity effect, thus creating a cluster of crystals around the optical fiber. Due to this, the presence of the fiber sensor enables the monitoring of the crystallization process of paracetamol.

After the addition of the paracetamol sample in (1), there is a decrease in optical power, as expected, as the surroundings of the sensor head are optically denser (higher refractive index compared to air refraction). With continued exposure to air in (2), the crystallization process is induced. The liquid-solid phase change occurs, presented in (3). At this phase, the sensor presents an unstable response, associated with the formation of crystalline structures around the sensor head which interfere with light reflection at the end of the MMF tip. At the end of the process of crystallization (4), the tip of the fiber sensor is surrounded by crystals, obtaining a response with some stability, as presented in Figure 7. Light is scattered in the presence of paracetamol crystals, where an effective index is considered, thus leading to an increase of the reflected signal intensity (4), although being lower than the intensity of the Fresnel reflection that resulted from the initial air/glass interface (1). Further, it is possible that between step (2) and step (3), the formation of some crystalline structures around the sensing head created an air cavity. For this reason, an intensity oscillation is observed between these two points. When the sensor is immersed vertically in the paracetamol solution, the liquid starts to fill this air cavity formed by the monoclinic polymorphs. Consequently, a fast oscillation in the intensity signal corresponding to the fast decrease in the length of the cavity, it is produced [27]. Assuming that higher oscillations of intensity correspond to higher viscosities and consequently slower fluid displacement, it can be recognized that even before step 3, the viscosity of the liquid changes, transforming this response into a signal oscillation. Further, the system allows measuring crystallization process regardless viscosity and flow velocity, as it depends on the acquisition rate (700 Hz, in this case).

## 4. Conclusions

In short, a refractometer sensor was developed for monitoring the paracetamol crystallization process. The sensing head proposed to combine the simplicity of its geometry and its implementation. In terms of practical sensing, this fiber tip configuration may provide in situ the monitoring of crystallization processes of active pharmaceutical ingredients (APIs) in the pharmaceutical industry, as the results showed that the sensing head is sensitive to the refractive index variations. Future developments may include this sensing head in a portable interrogation system to obtain a higher time resolution of the crystallization process of APIs.

## Supplementary Materials

The following are available online https://www.mdpi.com/1424-8220/20/1/87/s1.
